# Integrin Regulators in Neutrophils

**DOI:** 10.3390/cells11132025

**Published:** 2022-06-25

**Authors:** Sunitha Pulikkot, Liang Hu, Yunfeng Chen, Hao Sun, Zhichao Fan

**Affiliations:** 1 Department of Immunology, School of Medicine, UConn Health, 263 Farmington Ave., Farmington, CT 06030, USA; pulikkot@uchc.edu; 2 Academy of Integrative Medicine, Shanghai University of Traditional Chinese Medicine, 1200 Cai Lun Road, Shanghai 201203, China; fcchul@zzu.edu.cn; 3 Department of Biochemistry and Molecular Biology, University of Texas Medical Branch, 301 University Blvd., Galveston, TX 77555, USA; yunfchen@utmb.edu; 4 Department of Pathology, University of Texas Medical Branch, 301 University Blvd., Galveston, TX 77555, USA; 5 Department of Medicine, University of California San Diego, 9500 Gilman Drive, La Jolla, CA 92093, USA

**Keywords:** neutrophils, integrins, talin, RIAM, Rap1, kindlin

## Abstract

Neutrophils are the most abundant leukocytes in humans and are critical for innate immunity and inflammation. Integrins are critical for neutrophil functions, especially for their recruitment to sites of inflammation or infections. Integrin conformational changes during activation have been heavily investigated but are still not fully understood. Many regulators, such as talin, Rap1-interacting adaptor molecule (RIAM), Rap1, and kindlin, are critical for integrin activation and might be potential targets for integrin-regulating drugs in treating inflammatory diseases. In this review, we outline integrin activation regulators in neutrophils with a focus on the above critical regulators, as well as newly discovered modulators that are involved in integrin activation.

## 1. Introduction

Neutrophils account for ~60% of human circulating leukocytes, and they are central components of the host defense system for fighting against infections such as bacteria [1] and fungi [2]. Apart from their immune function against bacterial and fungal pathogens, their roles in antiviral host defense, such as human immunodeficiency virus (HIV) infection [3] and poxvirus [4], are also important.

Neutrophils are often considered effector cells of immune and inflammatory reactions due to their short-lived terminally differentiated nature [5] and effector functions, such as phagocytosis, respiratory burst, degranulation, or neutrophil extracellular trap (NET) release [6]. Mature neutrophils are technically challenging to study owing to their low mRNA content and sensitivity to environmental stimulations. Recent advancements in neutrophil biology, such as in vivo imaging, high-dimensional transcriptomic and epigenomic approaches, and studies performed at single-cell resolution, suggest that neutrophils serve functions that extend beyond their microbe-killing machinery. They respond to multiple biological signals to produce several cytokines and other inflammatory factors to regulate various functions in homeostasis and autoimmune disorders [7,8]. As heterogeneous neutrophils gained importance for their responses to different environmental challenges, determining their polarization and activation programs became critical.

**Figure 1:**
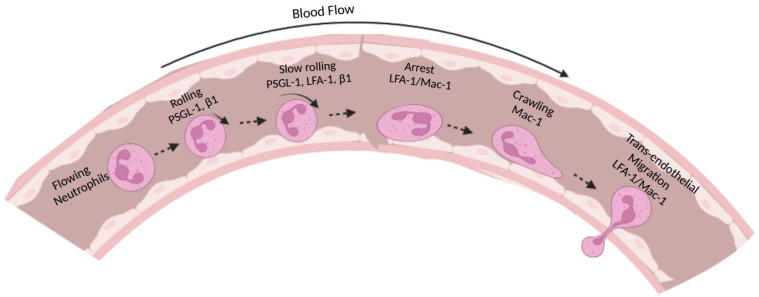
Schematic of neutrophil recruitment cascade: Flowing neutrophils roll on vascular endothelial cells through the interaction between PSGL-1 and selectins. Engagement of PSGL-1 with selectins induces the extension of LFA-1, which binds ICAM-1 or other ligands with an intermediate affinity and slows down rolling velocity. We call this process slow rolling. β1 Integrins are also involved in neutrophil rolling and slow rolling. When rolling neutrophils encounter chemokines, LFA-1 or Mac-1 will be fully activated and bind ICAM-1 or other ligands with a high affinity and stop rolling neutrophils. We call this process arrest. After arrest, neutrophils spread and crawl on endothelial cells, which is predominantly mediated by Mac-1. Neutrophils undergo trans-endothelial migration in an LFA-1/Mac-1-dependent manner to the tissue or site of injury. Created in BioRender.com (accessed on 12 June 2022).

Highly motile neutrophils leave the blood circulation for sites of infection through a recruitment cascade (Figure 1), which was described in previous reviews [5,9,10,11]. In the cascade, neutrophils first roll on the vascular endothelium by interacting with endothelial selectins using P-selectin glycoprotein ligand-1 (PSGL-1) [12,13]. When neutrophils encounter endothelial chemokines during rolling, integrins on neutrophils are activated, and neutrophils firmly adhere, which we call arrest [14]. After arrest, neutrophils can spread on the endothelium, perform trans-endothelium migration (TEM) [9], and migrate to the site of infection or inflammation [15,16]. Integrins play a central role in this cascade [17,18,19], as their activation and related signaling pathways mediate neutrophil arrest [20,21], TEM [9], and in-tissue migration to the site of infection or inflammation [15,16,22].

Integrins are well-studied molecules that belong to a family of transmembrane cell-surface adhesion receptors consisting of 24 transmembrane heterodimeric pairs that are generated from 18 α subunits and 8 β subunits by noncovalent association [23,24,25]. According to ligand specificity, they can be broadly separated into four groups: arginine-glycine-aspartate (RGD)-binding receptors, leukocyte-specific receptors, laminin-binding receptors, and collagen-binding receptors [26,27]. Both α and β subunits of the 700–1000 amino acid integrin extracellular domain form an elongated structure composed of a ligand-binding headpiece and a tailpiece [28,29,30,31]. Both integrin subunits have a single-pass transmembrane helical domain (∼20 amino acids), which interacts with two interfaces termed the outer and inner membrane clasps via hydrophobic interaction on the N-terminus and a salt bridge association on the C terminus. From the published data on integrin αIIbβ3, the outer membrane clasp is formed by G972/G976 in the αIIb subunit and G703 in the β3 subunit, and the inner membrane clasp is formed by F992/F993 and R995 in the αIIb subunit and W715/I719 and D723 in the β3 subunit [24,32]. The transmembrane domains are essential for integrin functions, as they allow bidirectional conformational changes. Transduction of biochemical signals and mechanical force across the plasma membrane requires the engagement of intracellular signaling and cytoskeletal proteins by integrin cytoplasmic tails and the engagement of extracellular ligands by integrin extracellular domains. This eventually disrupts the transmembrane domain interactions, separation, and angle changes of the transmembrane and intracellular domains [28,29,33,34]. Most integrin cytoplasmic tails are less than 75 amino acids in length, with the exception of β4, which is 1000 amino acids and contains four fibronectin type III repeats [35]. These repeats contain two NPXY (Asn-Pro-x-Tyr) motifs that can interact with phospho-tyrosine binding domains of intracellular proteins, such as talins [36], kindlins [37,38], and various other signaling and scaffolding molecules [39].

## 2. Types of Integrins Expressed on Neutrophils

Neutrophils express all four β2 integrins, including lymphocyte function-associated antigen 1 (LFA-1, also known as αLβ2 and CD11a/CD18), macrophage-1 antigen (Mac-1, also known as αMβ2, CD11b/CD18, and complement receptor 3), αXβ2 (also known as CD11c/CD18 and complement receptor 4) [40,41], and αDβ2 (also known as CD11d/CD18) [18,42], and five kinds of β1 integrins, such as α2β1 [43], α4β1 [44], α5β1 [45], α6β1 [46], and α9β1 [47].

β2 Integrins are leukocyte-specific integrins and are involved in most steps of the leukocyte recruitment cascade [48]. As mentioned above, the recruitment cascade is initiated with PSGL–selectin-mediated neutrophil rolling [12,13]. PSGL–selectin interaction induces the extension of β2 integrins, especially LFA-1 [49,50]. The extended low-affinity LFA-1 interacts with its ligand, intercellular adhesion molecule 1 (ICAM-1), and slows down the rolling velocity, which we call slow rolling [49,51,52]. Neutrophils encounter endothelial chemokines, such as interleukin-8 (IL-8) [53], or C-X-C Motif Chemokine Ligand 1 (CXCL1) [54], to initiate integrin inside-out signaling and fully activate β2 integrins so that they have high-affinity binding to ligands in trans, such as LFA-1 (both humans and mice) [49,50] and Mac-1 (humans but not mice) [12,13]. Fully activated β2 integrins bind endothelial ICAM-1 with high affinity and lead to neutrophil arrest [55,56]. Crawling through the inflamed vessel requires Mac-1 but not LFA-1. A Mac-1 knockout model resulted in crawling neutrophils with decreased distance and velocity [57]. It has also been shown that Mac-1 prevents neutrophils from migrating upstream, as their blockade allows neutrophil upstream migration in an LFA-1-dependent manner [58]. During transmigration, neutrophils move through the space in the disrupted endothelial membrane in a Mac-1 and LFA-1-dependent manner [59]. Neutrophils prefer the paracellular route (between endothelial cells) rather than the transcellular route (through endothelial cells) [22] through LFA1 binding to ICAM-1 [58,60,61,62], ICAM-2 [62,63,64], and JAM-A [64,65]. The release of myeloperoxidases by neutrophils during migration and their interactive products with the extracellular matrix (ECM) can, in turn, activate αMβ2 and αDβ2 integrins on macrophages at the site of inflammation [66]. Neutrophil β2 integrins play critical roles in other neutrophil functions, such as phagocytosis [40,41,67], cell differentiation [68,69], degranulation [70,71,72,73,74,75], and formation of NETs [3,76].

β1 Integrins are widely expressed on various cells, and some of them are expressed on neutrophils. They bind vascular adhesion molecules, such as vascular cell adhesion molecule 1 (VCAM-1), and components of ECM, such as fibronectin, collagen, and laminin [77]. Integrin α4β1 is expressed on rat and mouse neutrophils and mediates neutrophil adhesion to endothelial cells by interacting with VCAM-1 [47,78,79,80]. Human neutrophils may not express integrin α4β1 [44]. However, it has been shown that integrin α4β1 may be expressed on neutrophils from sickle cell disease [81] or sepsis patients [82] and contributes to neutrophil adhesion. It has been shown that human neutrophils adhere to laminin via an α6β1 integrin-dependent mechanism [46], to fibronectin via an α5β1-dependent mechanism [45], and to collagen via an α2β1-dependent mechanism [83]. Integrin α9β1 expression is upregulated two- to three-fold on human neutrophils after activation, and it binds VCAM-1 to stabilize interactions between rolling neutrophils and the endothelium and enhance β2 integrin-dependent neutrophil slow rolling and arrest. Neutrophil integrin α9β1 also binds to matrix proteins tenascin C and osteopontin [47]. Rodent studies reported the upregulation of α2β1 and α6β1 integrin membrane expression during neutrophil recruitment to inflammation sites [83,84,85,86]. The major role of β1 integrins is to mediate cell–matrix adhesion and promote leukocyte motility in the perivascular and ECM areas [87].

## 3. Integrin Conformational Changes during Activation

**Figure 2:**
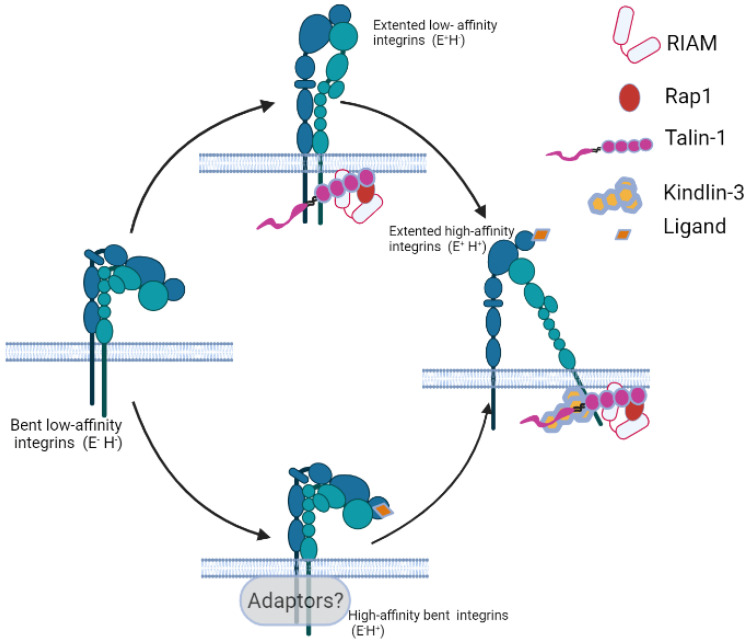
Schematic of the integrin activation conformational changes and key modulators: Resting integrins have a bent low-affinity (E^−^H^−^) conformation. They attain an extended low-affinity (E^+^H^−^) confirmation through the RIAM- and Rap1-mediated recruitment of talin-1. Further recruitment of kindlin-3 induces full integrin activation into extended high-affinity integrins (E^+^H^+^). There is an alternative allosteric pathway in which E^−^H^−^ integrins transition to a bent-high affinity conformation (E^−^H^+^) and then change to an extended high-affinity integrin (E^+^H^+^). Which integrin adaptors are involved in this allosteric pathway remains to be further investigated. Created in BioRender.com (accessed on 12 June 2022).

As mentioned above, integrins have long ectodomains, transmembrane domains, and short cytoplasmic tails [25,28,88]. They undergo large conformational changes in their extracellular domains during activation (Figure 2) to change their ability to bind ligands [25,89,90,91,92,93]. The ectodomain consists of a headpiece and a tailpiece. The conformational changes in integrins have been heavily discussed in previous reviews [20,25,34,42,66,90,94,95,96,97].

The canonical conformational change model in integrin activation is the switchblade model [25,49,96,98]. Resting integrins have bent extracellular domains, where their membrane-distal headpiece bends toward their membrane-proximal tailpiece [31]. In the switchblade model, bent low-affinity (E^−^H^−)^ integrins first extend to an extended low-affinity (E^+^H^−^) conformation. Then, the hybrid domain swing-out leads to high ligand-binding affinity in the headpiece and becomes an extended high-affinity (E^+^H^+^) conformation. However, the switchblade model does not cover the existing bent high-affinity (E^−^H^+^) conformation reported in αVβ3 [99], αXβ2 [100], and three Mac-1 mutants [101]. The identification of a ligand-bound bent-open integrin αVβ3 [31] suggested a deadbolt model [28]. This model proposes that a hairpin loop in the β tail domain acts as a deadbolt to restrain the displacement of the β-A/I domain β6-α7 loop and maintain the integrin in the H- state. The displacement of this loop releases to attain E^−^H^+^ conformation, allowing ligand binding with an auto-inhibitory effect on integrin αVβ3 in a binding complex with fibronectin activation. Then, the binding of ligands provides a pulling force that extends the ectodomain. This model is controversial in that the “deadbolt” works on Mac-1 [101] but not in β3 integrin [102]. Using live-cell quantitative dynamic footprinting microscopy, we observed both transitions of E^+^H^−^ to E^+^H^+^ and E^−^H^+^ to E^+^H^+^ in neutrophil β2 integrins, suggesting that both models may exist at the same time [102].

## 4. Integrin Activation Modulators

The integrin α and β chains contain conserved regions to bind different modulator proteins, such as NPXY motifs in the β cytoplasmic tail [39,103,104] and a GFFKR sequence in the α cytoplasmic tail [105]. Talins [106] and kindlins [37,107,108] are the most studied and well-accepted modulators that bind to the integrin β cytoplasmic tail and regulate integrin activation. Filamin A [109,110], DOK1 [111], 14-3-3ζ [112], α-actinin [113], Rap1-interacting adaptor molecule (RIAM) [114,115], and Rap-1 GTPases [116,117,118,119] were also reported to bind the integrin β cytoplasmic tail directly or indirectly. Calreticulin [120,121], RapL [122], paxillin [123], and SHARPIN [124] were reported to bind the integrin α cytoplasmic tail. SHARPIN also binds to the β cytoplasmic tail of β1 integrin [124]. There are some other molecules that reportedly modulate integrin activation. Whether they bind integrins directly is unknown. We discuss these integrin activation modulators in another section.

### 4.1. Talins

**Figure 3:**
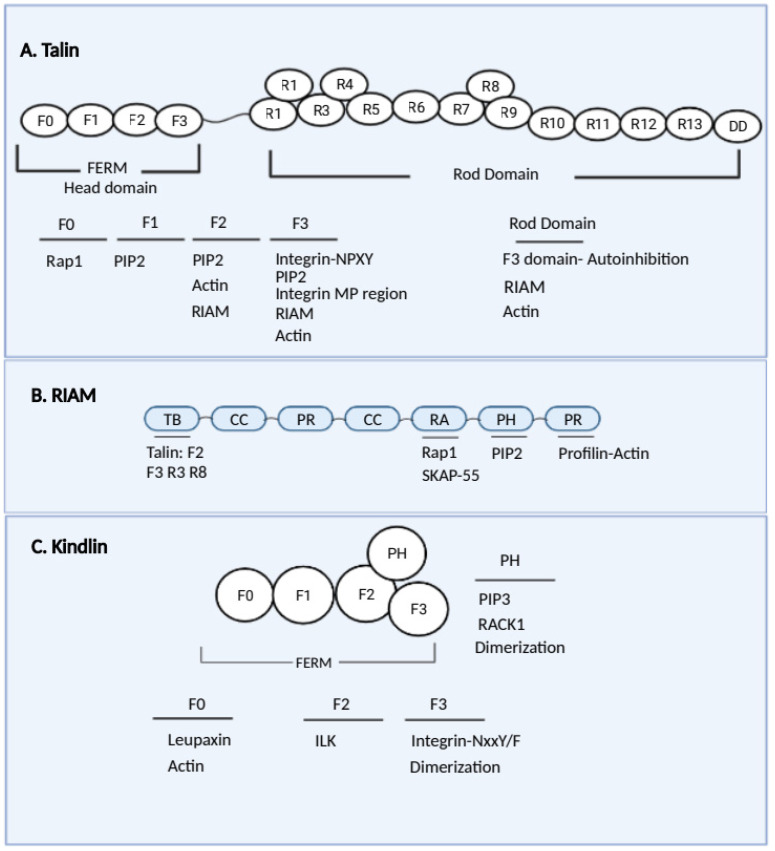
Schematics showing domains and binding proteins of talin (**A**), RIAM (**B**), and kindlin (**C**). Integrin-NPXY: Asn-Pro-x-Tyr motifs of the β-integrin tail; Integrin MP region: membrane-proximal region of the β-integrin tail; Integrin-NxxY/F: Asn-x-x-Tyr/Phe motifs of the β-integrin tail; PIP2: phosphatidylinositol 4,5-bisphosphate; PIP3: phosphatidylinositol-3,4,5-triphosphate; Profilin-Actin: direct binding to profilin and indirect binding to actin. Created in BioRender.com (accessed on 12 June 2022).

Talins are important integrin-binding proteins that exist in two isoforms, talin-1 and talin-2. Talin is composed of a 50 kDa N-terminal FERM (protein 4.1, ezrin, radixin, moesin) talin head domain (THD) and a 220 kDa C-terminal rod domain (Figure 3A) [125]. Talin-1 is expressed in all cell types, while talin-2 is highly expressed in skeletal and cardiac muscles and brain tissue [126,127]. A knockout study on mice showed that talin-1 is required for neutrophil LFA-1 extension and neutrophil slow rolling and arrest [128]. Talin-1-deficient mice showed defective neutrophil adhesion and spreading, along with impaired extravasation [119]. A knockdown study showed that talin-1 is required for Mac-1-mediated phagocytosis [129].

Both the THD and the rod domain have been reported to have integrin binding sites. The THD contains four subunits: F0, F1, F2, and F3 [130]. The F3 subunit of the THD interacts with the highly conserved membrane-proximal NPXY motifs of the cytoplasmic tails of integrin β subunits and induces integrin activation [131,132,133,134,135,136]. Mutations in the THD identified two sites that are involved in regulating integrin activation [136] and neutrophil adhesion [137]. The W359A mutation blocked talin interaction with integrins and showed similar neutrophil defects compared to the talin knockout. The L325R mutation did not block talin interaction with integrins or neutrophil slow rolling but showed a defect in neutrophil arrest and spreading. Mutations in the β2 integrin cytoplasmic tail showed that W747 and F754 are required for talin binding [129]. Talin recruitment to the integrin cytosolic tail is mediated by the GTPase Rap1 and RIAM (gene name: amyloid-beta precursor protein-binding family b member 1 interacting protein, APBB1IP) complex [118,138]. Recent works indicate that direct Rap1–talin1 binding plays a critical role in integrin activation in platelets [118,139,140] and also regulates integrin activation in blood cells [114,140,141]. The interaction of the THD F3 with the membrane-proximal α helix of the β integrin cytoplasmic tail disrupts the connection between cytoplasmic tails [142]. Consecutive electrostatic interactions between the THD and phospholipids on the plasma membrane contribute to the separation and re-orientation of integrin cytoplasmic tails [143]. The THD F3 forms a well-defined complex with the helix-forming membrane-proximal (MP) region of the β-integrin tail, and this interaction holds the key to the molecular recognition required for activation [136]. The talin rod contains a second integrin-binding site (IBS2) that may bind the membrane-proximal region of the integrin cytoplasmic domain, which may only happen after integrin activation [144,145,146].

A resting talin is autoinhibited. The integrin-binding sites of talin are masked in its autoinhibitory conformation, where the rod segment folds back onto its F3 subdomain [36]. Autoinhibition of talin has been shown to regulate its recruitment to adhesome sites and the maturation of focal adhesions in mouse embryonic fibroblast cells [147]. THD binding to the rod inhibits its binding to the integrin cytoplasmic tail. There are interactions between talin molecules to form dimers, showing an inhibitory effect on talin [148]. It has been shown that in β1 integrins, a mutation that blocks talin autoinhibition leads to increased integrin activation in mouse embryonic fibroblast cell lines, along with increased focal adhesion maturation and stability [147]. Phosphatidylinositol 4,5-bisphosphate (PIP2) binding to talin F2 and F3 domains [149], calpain cleavage [150], and phosphorylation events [147,151] can activate talin. However, these have not been demonstrated in neutrophils.

Although the F3 domain forms a complex with integrin, F0 and F1 domains are also required for integrin activation. Although talin F3 can bind β1A and β3 tails with similar affinity on CHO or HT1080 cells (FN9–11 binding), the expression of the whole talin head (residues 1–405) is required for β1A-integrin activation. The activation especially requires the F0 F1 domain, in which the N-terminal residues 1–85 precede the FERM domain (F0 domain), the F1 FERM domain, and the integrin-binding F3 domain [152]. A large loop within the F1 domain shows that it has a propensity to adopt a helical structure in which basic residues are clustered on one surface and that it interacts with vesicles containing acidic phospholipids [153]. These findings have not been demonstrated in neutrophils as well.

### 4.2. RIAM

RIAM is a member of the Mig-10/RIAM/Lamellipodin (MRL) protein family (Figure 3B), which is directly bound to talin via a short N-terminal sequence that was predicted to form amphipathic helices. RIAM binds Rap1, which is discussed in a later section, and functions as a scaffold that connects the membrane-targeting sequences in Ras GTPases to talin [138]. The inhibitory (IN) segment of RIAM and the RA domain at its Rap1-binding site form an autoinhibitory conformation to lock RIAM in an inactive state. Phosphorylations of the IN segment and PH domain by FAK and Src, respectively, release the inhibitory state and activate RIAM, facilitating RIAM interaction with Rap1 and PIP2 to induce talin-1-dependent integrin activation [154,155]. RIAM-induced integrin activation requires its capacity to bind to both Rap1 and talin [115]. Interestingly, RIAM and vinculin have mutually exclusive binding sites on talin [156]. In adhesive cells, RIAM-containing adhesions are primarily in the lamellipodium. RIAM is subsequently reduced in mature focal adhesions due to direct competition with vinculin for talin-binding sites [157]. Whether it has a similar mechanism during neutrophil migration remains to be investigated.

RIAM is abundant in hematopoietic cells, and its absence blocks agonist-mediated αIIbβ3 activation in primary mouse megakaryocytes [158] without affecting development, homeostasis, or platelet integrin functions, as RIAM levels are low in normal platelets, whereas Rap1, talin1, and integrins are highly expressed in platelets, indicating the existence of RIAM-independent Rap1 regulation [159]. RIAM has an indispensable role in the activation of β2 integrins in neutrophils, macrophages, and T cells [119,160]. Studies conducted on mice that both carry Rap1-binding mutant talin1 and lack RIAM expression showed increased neutrophil rolling velocities and decreased adhesion to inflamed cremaster muscle venules compared to wild type or single-mutant mice (Rap1-binding mutant talin1 knock-in or RIAM knockout) by affecting conformational changes in the β2 integrin ectodomain [114]. This specifically regulates leukocyte β2 integrins [119]. Extravasation, αMβ2-mediated adhesion, and spreading to immobilized immune complexes were found to be impaired in RIAM knockout neutrophils [119]. RIAM mainly regulates the activation of β2 integrins; however, it only partially affects integrin β1 [119]. Moreover, RIAM is dispensable for integrins in other cell types, such as fibroblasts or platelets. This may be because (1) the expression of RIAM is low in these cells; (2) the RIAM-dependent β2 activation complex is only formed in certain hematopoietic cells [114]. The hypothesis is supported by recent work that showed that RIAM is dispensable for β2 integrin activation in regulatory T cells [97]. In regulatory T cells, β2 integrins can be activated by Lamellipodin, which is the RIAM paralog protein and is highly expressed in regulatory T cells in the absence of RIAM. These results revealed that RIAM function differs depending on the cell type and integrin class, suggesting a potential method to specifically manipulate the trafficking and function of selective cell types.

Adaptor molecules such as 55 kDa src kinase-associated phosphoprotein (SKAP-55) and the adhesion and degranulation-promoting adapter protein (ADAP) consistently interact with RIAM to promote the membrane targeting of the RIAM-Rap1 module for antigen stimulation of T cells to facilitate LFA-1 integrin activation [161].

### 4.3. Rap1

Rap1 proteins are small GTPases of the Ras family and are encoded by two Rap1 genes, Rap1A and Rap1B [162]. GDP-bound inactive Rap1 is activated when GTP is exchanged for GDP, which is regulated by several guanine nucleotide-exchange factors (GEFs), and they participate in various signaling pathways, including integrin activation, ERK activation, and other effector pathways [163]. Rap1 signaling is terminated by the hydrolysis of bound GTP to GDP by specific GTPase-activating proteins (GAPs) such as Rap1GAP1 [164] and signal-induced proliferation-associated gene-1 (SPA-1) [165]. Activation of Rap1 GTPase effectors was found to have different consequences on various cells, which has been reviewed before [166,167]. They have a wide variety of functions, such as control of the establishment of cell polarity [91,168], activation of integrin-mediated cell adhesion [169,170], and the regulation of cell–cell contacts [171,172], migration [172,173], cell proliferation [174], and secretion [163,166]. Rap1 deficiency can markedly suppress neutrophil functions by inhibiting the activation of β2 integrin [118].

Despite the Rap1/RIAM/talin axis for integrin activation [114,138], the membrane-anchored Rap1 binds to the F0 domain of talin [118,139]. Although the binding of Rap1 and the talin F0 domain is critical in *Drosophila* [139,175], it displayed mild defects in talin1-induced αIIbβ3 activation and platelet function, including aggregation and hemostasis [139]. A novel Rap1-binding site in the talin F1 domain was recently identified [176]. Blocking the ability of Rap1 to bind to the talin F1 domain profoundly disrupted the Rap1 function that mediates talin1-induced integrin activation in platelets [140,176]. The association of Rap1 and the talin F1 domain plays a central role in Rap1-mediated integrin activation in platelets [140,177]. Moreover, recent work reported that the Rap1 and talin association plays a critical role in integrin activation in leukocytes as well [114,178]. The Tln1^3mut^ mouse with K15A, R30A, and R35A mutations in the F0 domain showed a mild neutrophil adhesion defect and reduced extravasation [118]. However, the Tln1^3mut^ mutation combined with RIAM deficiency dramatically blocked neutrophil integrin functions [114]. However, the talin F1 mutation R118E, which blocks the binding of Rap1 to the talin F1 domain, significantly affected both neutrophils and lymphocytes [140,178], indicating that Rap1 binding to talin F1 plays a more important role than the Rap1–F0 interaction. The role of direct Rap1–talin binding in neutrophil function warrants further investigation.

The RASGRP2 gene encodes the Ca^2+^ and diacylglycerol-regulated guanine nucleotide exchange factor I (CalDAG-GEFI) protein, a guanine nucleotide-exchange factor for the small GTPase Rap1; this is indispensable for platelet Rap1 activity, as mutations in this gene abrogate Rap1 activation and cause platelet dysfunction [179]. This gene is highly expressed in neutrophils and also showed defective integrin activation in mutational studies [180]. Neutrophils from CalDAG-GEFI knockout mice exhibited strong defects in Rap1 and β1 and β2 integrin activation while maintaining normal calcium flux, degranulation, and ROS generation. Hence, neutrophils in these mice failed to adhere firmly to stimulated venules and migrate into sites of inflammation [180].

The PH domain of SWAP-70-like adaptor of T cells (SLAT), also known as DEF6, has a regulatory function on the active form of the small GTPase Rap1 in LFA-1 activation on T cells [181]. Similarly, B cell adaptor molecule of 32 kDa (Bam32) has a suppressive role in chemokine-induced neutrophil chemotaxis and transmigration by regulating Rap1 activation in neutrophils [182].

A recent study reported that phostensin (PTSN), a regulatory subunit of protein phosphatase 1, mediates dephosphorylation of Rap1 and regulates integrin activation [183]. PTSN is mainly expressed in leukocytes [184], and its tissue distribution is similar to that of RIAM. PTSN-deficient mice exhibited a significant increase in peripheral blood neutrophils, suggesting that PTSN may play an important role in modulating neutrophil function [183].

### 4.4. Kindlin

Kindlin is another integrin-regulating protein that contains a 4.1-ezrin-radixin-moesin (FERM) domain and a pleckstrin homology (PH) domain (Figure 3C). There are three kindlin paralogs, kindlin-1, -2, and -3. Kindlin-1 is mainly expressed in epithelial cells and keratinocytes. Kindlin-2 is expressed in many cell types, such as muscle cells and epidermal cells, but not in hematopoietic cells [185]. Kindlin-3 is expressed in all hematopoietic cells [186]. Kindlin-1 binds integrins β1, β3, and β6, and kindlin-2 and -3 bind integrins β1, β2, and β3 [187] to regulate integrin activation [188].

Mutations in the kindlin-3 gene *FERMT3* cause LAD-III syndrome [189,190]. In a mouse knockout study, kindlin-3 was found necessary for the LFA-1 high-affinity conformation and neutrophil arrest [128,191,192]. The FERM-like kindlin molecule contains the F0, F1, F2, and F3 subdomains linearly with a unique PH domain inserted into the F2 subdomain [193,194]. The kindlin-3 F3 subdomain binds to the distal NxxY/F motif [188] and the TTT^759−761^ motif [195] of the integrin β2 cytoplasmic tail. Kindlin-3 binds β2 integrin through its QW^614/615^ residues in its F3 subdomain, and a mouse strain carrying the QW/AA mutation was generated [196]. Neutrophils from this mouse strain showed defects in both adhesion and NET release [197]. However, a follow-up study from the same group showed that overexpression of kindlin-3, regardless of WT or QW/AA mutation, in neutrophil-like HL60 cells inhibited NET generation [198]. Kindlin-3 knockdown HL60 cells and neutrophils from kindlin-3^flox/flox^Mx1-Cre mice showed enhanced NET generation [198].

Kindlin-3 is also important for podosome assembly by regulating integrin activation and clustering [199]. Furthermore, the kindlin-3 F0 domain binds leupaxin, recruits leupaxin into podosomes, dephosphorylates paxillin, and increases podosome stability [199]. Kindlin-3 was recruited to the plasma membrane in response to interleukin-8 (IL-8) before the induction of high-affinity β2-integrin conformations [107,200,201]. The PH domain of kindlin is necessary for the plasma membrane recruitment of kindlin-3, which occurs before rolling. The PH domain of kindlin-3 interacts with the scaffold protein receptor of activated protein C kinase 1 (RACK1) [202]. A highly conserved poly-lysine motif in the loop of the domain of kindlin-1 [203] and kindlin-3 [204] supports binding to the negatively charged phospholipid head group. A recent study showed that the F2 PH and F3 subdomains are important for kindlin self-association, which negatively regulates integrin binding, and they identified kindlin-3 point mutations that decrease self-association and enhance integrin binding while maintaining the ability to localize to focal adhesions [24,205].

In addition to integrins, kindlin proteins also interact with binding partners such as integrin-linked kinase (ILK), migfilin, and RACK1 [202]. ILK is reported to have a PKCα-mediated phosphorylation function on kindlin-3, which is required for the chemokine-induced full activation of LFA-1 [206].

### 4.5. Linking of Integrins to the Actin Cytoskeleton

Linking integrins to the actin cytoskeleton is important for force transmission from extracellular ligands to neutrophil cytoskeletons to support neutrophil adhesion and migration. Moreover, treatment with latrunculin B or blebbistatin to block actin polymerization in human neutrophils impaired β2 integrin activation to high-affinity conformations and inhibited human neutrophil arrest on ICAM-1 [207]. Thus, knowing how the actin cytoskeleton tethers to leukocyte integrins is important in understanding β2 integrin function.

Several integrin activation modulators mentioned above, such as talin, RIAM, and kindlin, have actin-binding sites. Talin (Figure 3A), the THD, and rod domains contain direct actin-binding sites and indirect sites that bind actin-binding vinculin, etc. [125,142,207,208,209]. The F0 domain of kindlin-2 (Figure 3C) was reported to bind actin directly [210]. RIAM (Figure 3B) binds actin indirectly through profilin [211].

α-Actinin is a cytoskeletal actin-binding protein that binds to β2 integrins directly [212]. The cytoplasmic tails of the integrin β2 subunit bind to α-actinin in focal adhesions, adherent junctions, and hemidesmosomes [213]. In fibroblasts, talin and α-actinin compete for binding to β3 integrins [214]. Deletion of α-actinin enhances force generation in initial adhesions on fibronectin but impairs mechanotransduction in a subsequent step, preventing adhesion maturation [214]. It has been shown that α-actinin may be expressed in neutrophils [215,216]; however, the role of α-actinin in regulating neutrophil integrins remains unknown.

Coronin 1A is a family member of evolutionarily conserved actin-binding proteins that regulate actin cytoskeleton-dependent processes. It is predominantly expressed in leukocytes [217] and has been identified as a regulator of β2 integrins that interacts with the cytoplasmic tail of β2 integrins and is crucial for the induction of neutrophil adhesion, spreading, and migration [218]. Coronin 1A knockout neutrophils showed less β2 integrin-dependent soluble ICAM-1 binding and LFA-1 clustering. Another study showed that loss of Coronin 1A results in less β2 integrin translocation to the platelet surface [219].

Transient receptor potential channel 6 (TRPC6) knockdown showed attenuated chronic hypoxia-induced actin assembly and actin reorganization in human mesangial cells [220]. TRPC6 has a significant role in the CXCL1-dependent recruitment of murine neutrophil granulocytes. The recruitment of neutrophils after renal reperfusion injury was attenuated in TRPC6 knockout mice. TRPC6 knockout neutrophils showed diminished Rap1 and β2 integrin activation, resulting in decreased CXCL1-induced neutrophil adhesion and transmigration [221].

### 4.6. Other Molecules That Regulate Neutrophil Integrins

Mitofusins (MFNs) are GTPases embedded in the outer membrane of the mitochondria and are essential for mitochondrial fusion. It was found that interfering with MFN2 expression using shRNA can significantly suppress the chemotaxis of neutrophil-like DMSO-differentiated HL-60 (dHL60) cells [222]. This was further demonstrated by neutrophil recruitment defects in MFN2 knockout zebrafish and MFN2^flox/flox^MRP8-cre mice [223]. MFN2 knockdown dHL60 cells showed a defect in adhesion on inflamed human umbilical vein endothelial cells (HUVEC) [223]. Using microfluidic devices, our group has shown that MFN2 knockdown dHL60 cells have defects in slow rolling and arrest on P-selectin/ICAM-1 and P-selectin/ICAM-1/IL8 substrates, respectively, suggesting a role of MFN2 in regulating β2 integrin activation on neutrophils [224]. MFN2 knockdown dHL60 cells have reduced formyl peptide receptor (FPR) expression and FPR-dependent (fMLP stimulation) and independent (PMA stimulation) β2 integrin activation defects. MFN2 knockdown dHL60 cells also show defects in actin polymerization after fMLP or PMA stimulation and Mn^2+^-induced cell spreading on ICAM-1. We demonstrated that MFN2 knockdown HL60 cells are unable to differentiate into neutrophil-like dHL60 cells by assessing the nuclear morphology and maturation markers CD35 and CD87. Using the CD87 maturation marker, we found that in the mature CD87high population, MFN2 knockdown HL60 cells still show defects in cell slow rolling, adhesion, and β2 integrin activation, indicating that, besides its effects on differentiation, MFN2 is directly involved in regulating β2 integrin activation. Please note that in these mature populations, MFN2 only affects extension (which is reported by the KIM127 antibody) but not headpiece opening (which is reported by the mAb24 antibody) under PMA stimulation. This suggests that MFN2 might be important for the conformational changes of bent-open to extended-open β2 integrins, which is an alternative allosteric pathway of β2 integrins that we observed before [21,102,224,225,226], in addition to the conical switchblade model [25]. However, since MFN2 is an important mitochondrial regulator, whether MFN2 directly affects the integrin activation pathway or indirectly affects integrin activation by altering mitochondrial function is unclear. It has been shown that MFN2 is critical for mitochondrial respiration in fibroblasts [227] and macrophages (Tur et al., 2020). Whether it is the same in neutrophils is unknown. MFN2 is also known to regulate the tethering of mitochondria and endoplasmic reticulum, which is important for intracellular calcium regulation [228,229]. It has been shown that MFN2 is involved in mitochondria–endoplasmic reticulum interaction in neutrophil-like HL60 cells and may regulate intracellular calcium [223]. Thus, it is also possible that MFN2 regulates integrin activation through intracellular calcium. In macrophages, MFN2 silencing leads to reduced ER–mitochondria contacts and mitochondrial activity. The inflammatory and pro-inflammatory responses upon administration of inflammatory agents also showed increased responses in MFN2-deficient macrophages [230]. Deficient ROS production in the absence of MFN2 impairs the induction of cytokines and nitric oxide and is associated with dysfunctional autophagy, apoptosis, phagocytosis, and antigen processing. The lack of MFN2 in macrophages causes an impaired response in a model of non-septic inflammation in mice, as well as a failure in protection from *Listeria*, *Mycobacterium tuberculosis*, or LPS endotoxemia [231,232].

Several cytoplasmic components regulate the interactions of integrin through the process of phosphorylation and dephosphorylation. Leukocyte integrin α chain phosphorylation at Ser-1140 (αL), Ser-1126 (αM), or Ser-1158 (αX) is essential for leukocyte adhesion and intracellular signaling [233,234,235]. The β2 chain becomes phosphorylated after activation through chemokines, the TCR, or phorbol esters. The protein kinase C (PKC) enzyme phosphorylates the β2 chain at Thr-758, leads to the release of bound filamin, and promotes the binding of 14-3-3 proteins, whereas talin can bind to both the Thr-758 phosphorylated and unphosphorylated chains [112]. Following T cell receptor stimulation, phosphorylation of the LFA-1 β2 chain on Thr-758 leads to 14-3-3 recruitment to the integrin, actin cytoskeleton reorganization, and increased adhesion [236]. Another study conducted using a phosphorylation model on the LFA-1 α chain at Ser-1140 showed that it affects β chain phosphorylation at Thr-758 with significantly reduced binding of α-actinin and 14-3-3 on SDF-1α-activated mutant cells [113].

Dok1 and Dok2 negatively regulate immune cell signaling [237], and Dok1 specifically negatively regulates the Ras-ERK pathway by binding to p120RasGAP [238]. Dok1 is an integrin inhibitor that competes with talin for binding to the tyrosine-phosphorylated proximal NPXY sequence in β3 [106]. Dok1 and Dok2 bind weakly to β2 cytoplasmic peptides but bind strongly to peptides phosphorylated at S756. In the neutrophil αMβ2 integrin, the small G protein Rap1 binds to the phosphorylated S756 and blocks Dok1 binding-mediated integrin inhibition [239].

Shank-associated RH domain-interacting protein (SHARPIN) is a 45 kDa cytosolic protein that is widely expressed [240]. It is one of the three subunits of the linear ubiquitin chain assembly complex (LUBAC), an E3 ubiquitin ligase enzyme complex [241,242]. This complex comprises two subunits other than SHARPIN: a large isoform of heme-oxidized iron regulatory protein 2 (IRP2) ubiquitin ligase 1 (HOIL-1L) and HOIL-1L interacting protein (HOIP), on which the RING-in-between-RING (RBR) domain of HOIP is the catalytic center for linear ubiquitination [243,244]. A high-throughput RNAi screen in PC3 prostate cancer cells identified SHARPIN as an endogenous inhibitor of β1-integrin activity. SHARPIN silencing induced a significant increase in active β1 integrins on the cell surface without altering total surface β1 integrin expression [245]. SHARPIN could inactivate integrins by modulating the expression and/or function of the β1-integrin activators talin or kindlin or via the LUBAC-stimulated formation of linear ubiquitin chains involved in signaling [246]. SHARPIN regulates β1 integrin-dependent cell adhesion and migration through its unique binding to the β1-associated α chain and by inhibiting the recruitment and binding of kindlin and talin to the integrin in several normal and malignant cell types [124]. The loss of SHARPIN and increased integrin activity in mice in vivo depicts its negative regulation in integrin activation [245]. Mice deficient in SHARPIN had increased neutrophils in the spleen and peripheral blood [247]. However, whether it is due to global inflammation in these mice or a cell-intrinsic abnormality of integrin activation in neutrophils requires further investigation.

## Data Availability

Not applicable.
